# Radiographic evaluation of gastroliths in a group of 49 juvenile saltwater crocodiles (*Crocodylus porosus*) in the UK

**DOI:** 10.1002/vetr.70213

**Published:** 2026-01-04

**Authors:** Charlotte R. Nix, Ellie R. John, Iain Cope, Katherine Hughes, Marie‐Aude Genain

**Affiliations:** ^1^ Department of Veterinary Medicine The Queen's Veterinary School Hospital, Cambridge University Cambridge UK; ^2^ Vets4Pets Newmarket Suffolk

**Keywords:** diagnostic imaging, exotics, radiography, reptiles, zoo animals

## Abstract

**Background:**

Gastrolith ingestion is a known inherent behaviour in crocodiles. However, the interpretation of coelomic radiographs in crocodilians is challenging due to the limited informative literature in this field.

**Methods:**

Dorsoventral coelomic radiographs of 49 juvenile saltwater crocodiles (*Crocodylus porosus*) were acquired twice, with an interval of 4 months. Any gastroliths present were evaluated and classified as sand, small, medium and large, relative to the size of the crocodile, by comparing the gastrolith size to the length of the 11th dorsal vertebral body.

**Results:**

A total of 1971 gastroliths were recorded during the two examinations (mean of 21 per crocodile, range 0‒98). Small gastroliths were the most commonly recorded (94%). There was no significant correlation between weight gain and gastrolith numbers in either examination (*p* = 0.506 and 0.2034). There was a significant reduction in small gastroliths (*p* = 0.034) and a significant increase in medium gastroliths (*p* = 0.023) between examinations.

**Limitations:**

All radiographs were performed on a cohort of juvenile crocodiles at the same collection. Orthogonal lateral radiographic projections were not performed due to practical and ethical limitations.

**Conclusions:**

Large numbers of relatively small gastroliths are likely a normal radiographic finding in juvenile crocodiles. As crocodiles increase in size, ingestion and retention of medium/large gastroliths may reduce body buoyancy to aid in increasing dive times and hunting of larger prey.

## INTRODUCTION

Captive rearing of crocodilians has been reported since the early 20th century for conservation, public education and as a sustainable method of producing meat and skin for international trade.^1^, ^2^ Rearing saltwater (*Crocodylus porosus*) crocodiles is known to be challenging, with reported early survival probabilities of around 51% to day 400 in captivity,^1^, ^3^, ^4^ increasing to greater than 95% up to 3‒5 years of age.^3^, ^4^, ^5^, ^6^ Lower average survival rates (30%‒66%) are reported in wild salt‒water crocodile populations due to predation.^5^, ^7^ The most commonly reported causes of death in juvenile crocodiles are infectious disease^8^, ^9^ and ‘runting’, a term that describes poor growth in young juvenile crocodiles with wasting body condition and early deaths.^6^, ^10^, ^11^

Foreign body ingestion remains an important differential diagnosis for individual crocodiles presenting with clinical signs of gastrointestinal disease, including anorexia, polyphagia, pica, cloacal prolapse, vomiting, diarrhoea, constipation, lethargy and weight loss.^12^, ^13^, ^14^ Crocodiles and alligators are known to have an inherent behaviour of deliberately ingesting mineral material both in the wild and in captivity.^15^, ^16^, ^17^, ^18^, ^19^ The behaviour of ingesting mineral material, termed ‘lithophagy’, has been demonstrated in juvenile crocodiles from an early age.^18^, ^19^ Unfortunately, mineral is not the only material crocodiles have a tendency to ingest, with several reports describing surgical or endoscopic removal of other types of foreign material in crocodiles in the wild and captivity.^20^, ^21^, ^22^, ^23^, ^24^ Malfunction of water pumps/filters, construction or visitor pollution (coins) in captivity can lead to the ingestion of sharp or metallic foreign bodies, which may cause toxicosis or coelomitis.^12^, ^16^

Radiographs of the coelom are often performed as part of the diagnostic approach for evaluating the presence of radiopaque foreign material or pneumocoelom secondary to gastrointestinal perforation. If free coelomic gas is not present, this may create a diagnostic challenge, as it may be difficult to determine whether a radiopaque foreign body is clinically significant or an incidental finding. Although many studies have demonstrated the presence of gastroliths in crocodiles, there is limited literature describing what is likely within normal limits regarding the quantity and size of gastroliths. A search of the literature using keywords ‘gastrolith’ and ‘alligator’ or ‘crocodile’ revealed no previously published studies evaluating gastroliths radiographically in a normal crocodilian population.

This study focused on a collection of juvenile saltwater crocodiles in the UK with a history of previous crocodile mortalities associated with toxicosis from metal foreign bodies (coins) and coelomitis from sharp objects. The ingestion of metal or plastic has been identified as a common problem in both captive and wild crocodilian populations.^16^, ^17^ This population was therefore considered representative for the objectives of this study. The primary objective of this study was to evaluate the number and size of radiopaque gastrointestinal foreign bodies in a group of juvenile crocodiles to establish what is likely a normal finding. The secondary objective of this study was to investigate any correlation between the weight of the crocodiles and the size or amount of gastroliths over time.

## METHODS

A prospective observational study was conducted in a UK zoo, with a full zoo license, between June and October 2023. A cohort of 49, 2‐year‐old, juvenile saltwater crocodiles (*C. porosus*), imported from Borneo in December 2022, was initially included in the study. All protocols involving animals included in this study have been reviewed and approved by the Departmental Ethics and Welfare Committee at the Department of Veterinary Medicine, University of Cambridge (CR672). The cohort was housed together as a group in a single indoor enclosure that consisted of an artificial pool (tub) and a dry land area. Rocks, sand and vegetation were provided to simulate their native habitat. The crocodiles were provided with natural sunlight and additional reptile D3 basking ultraviolet (UV) lamps that emit heat, UVA and UVB. During the study, the UV lamps were positioned at a height of 2 m as per the manufacturer's guidelines. The humidity of the enclosure was maintained at 70%‒80%. The air temperature, water temperature and heat‐emitting UV lamps were carefully controlled to provide the crocodiles with an appropriate temperature gradient, compliant with published guidelines.^25^ Air temperatures were maintained at 27°C‒30°C, dependent on seasonal variability due to natural sunlight. In the warmer months (June), the pool temperature was maintained between 25°C and 27°C, with air temperatures ranging from 28°C to 33°C. In the colder months (October), extra heat was provided through the UV lamps, increasing the pool temperature to 30°C. The water supplied in the pools was appropriately aerated and filtered. The average length of the crocodiles during the entire examination (E1 and E2) was 120 cm. The cohort of crocodiles was offered a consistent diet during the examination period, fed every other day, following a feeding chart based on the crocodile's average length, compliant with published feeding recommendations.^26^ The crocodiles were fed an average of 380 g of meat per crocodile per feed. The crocodiles were fed every other day during the examination time period, with an average food intake of 1140 g per week. The meat comprised mostly of poultry carcasses (including bones) and pig hearts, with calcium supplementation (Calvi‐Dust D3; Vetark) according to the manufacturer's guidelines. The gut‐loaded insects were provided occasionally on days between feeds to provide stimulation and extra vitamin supplementation. During feeding, each animal was visually monitored to ensure that the food was distributed appropriately, with directed feeding performed when required. If an animal did not feed appropriately after two feeds, then it was notified to the zoo veterinarian and placed under medical observation.

Examination 1 (E1) was performed in June 2023. In order to reduce handling stress, the procedure was timed to coincide with planned movements of the crocodiles to a new enclosure or for medical checks.

### Radiographic acquisition

The saltwater crocodiles were individually captured and placed, un‐sedated, in a plexiglass and wooden box where a dorsoventral radiographic projection of the crocodile's coelomic cavity was obtained. Manually restrained or horizontal beam orthogonal lateral radiographic projections were not performed in this study due to the radiation safety on the farm, the capacity of the plexiglass box (only containing plexiglass on the top and bottom to ensure stability) and ethical concerns of inducing unnecessary stress by excessive handling and restraint of the crocodiles.

A portable digital X‐ray machine (MeX+20BT Lite, XPrime) was used to acquire the radiographs, with exposure values between 60 and 75 kVp and between 4.5 and 6.4 mAs, which were manually adjusted depending on the size of the crocodile. No grid was used in this study. Each crocodile was identified by its microchip number, and its weight, size (neck and tail width, total length) and body condition score (0‒5) were obtained as established in previous studies.^27^ Examination 2 (E2) was performed 4 months later, where the whole procedure was repeated.

### Radiographic evaluation

**FIGURE 1:**
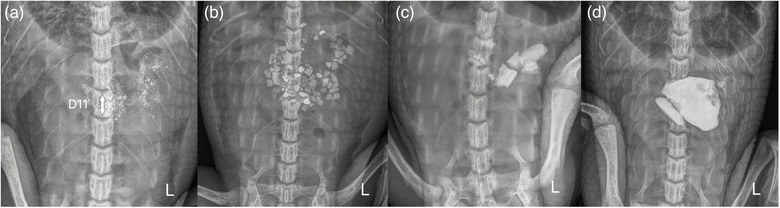
Dorsoventral radiographs of the coelomic cavity of crocodiles containing different relative sizes of gastrolith, measured with respect to the length of the dorsal 11th vertebrae (D11) (white arrow). (a) Sand and six small gastroliths; (b) 87 small gastroliths (<0.5 × length of D11); (c) seven small gastroliths (>0.5 × length of D11), three medium gastroliths (0.5‒1 × length of D11) and one large gastrolith (0.5‒1 × length of D11); and (d) two large gastroliths (>1 × length of D11) and five small gastroliths (<0.5 × length of D11).

The radiographs were evaluated using a medical imaging software^28^ by a veterinary radiology resident (C.R.N.), supervised by an ECVDI diplomat (M.‐A.G.), at The Queen's Veterinary School Hospital, Cambridge. The resident was blinded to the identity and weight of the crocodile during the radiographic analysis. The gastrointestinal tract was evaluated for the presence and number of mineral gastroliths. Crocodiles have nine cervical vertebrae, 15 dorsal vertebrae, two sacral vertebrae and a varying number of caudal vertebrae,^29^ totalling 26 precaudal vertebrae. The length of each vertebrae (centrum) has been shown to vary depending on the breed, age and maximal mature size of the crocodile.^30^ The vertebral length was selected as a measurement tool to evaluate the relative size of the gastroliths when compared with the size of the crocodile to enable veterinarians to utilise this method when evaluating radiographs of crocodiles of different ages, sizes and species. A small non‐obstructive gastrolith for a large crocodile may be a large obstructive gastrolith for a small crocodile. The relative size of the gastroliths in this study was compared to the length of the 11th dorsal vertebral body (D11) as this was the most centrally positioned vertebral body with the least amount of superimposition from gastroliths. Very small mineral material was recorded as sand (Figure 1A). The presence of sand was given a numerical stone value of 1, as it was not possible to count the number of grains of sand. The remaining gastroliths were counted and recorded in relative size categories as small (<0.5 × length of D11), medium (0.5‒1 × length of D11) and large (>1 × length of D11) (Figure 1).

### Pathological examination

Four crocodiles that died within the time frame between the two radiographic studies were submitted for routine diagnostic postmortem examination or were frozen and underwent a targeted postmortem examination. For histopathology, representative pieces of selected tissues were fixed in 10% neutral buffered formalin. These selected tissue samples were subsequently processed and sectioned using routine histological protocols. Staining with haematoxylin and eosin, periodic acid‐Schiff, Gram and modified Ziehl‒Neelsen also followed standard protocols.

### Statistical methods

The statistical analysis was performed by a professional statistician (E.R.J.). Descriptive statistics were used to summarise the characteristics recorded at E1 and E2, respectively. Continuous variables were summarised using the means and standard deviations alongside the medians and interquartile ranges. Categorical variables were summarised using numbers and percentages within each category. All continuous variables were plotted on a Q‒Q plot to assess normality and demonstrated a broadly normal distribution with no significant deviations. All continuous variables were compared using a *t*‐test. A *p*‐value of less than 0.05 was considered significant. No adjustment for multiple testing was applied. The change in weight of each crocodile was calculated by subtracting the E1 weight from the E2 weight. E1 characteristics were compared between alive crocodiles at E2 and those that were deceased at E2. A separate analysis, excluding patients deceased at E2, was performed to evaluate changes in the number of gastroliths and weight between the two examinations. All analyses were conducted using R statistical analysis software.^31^

## RESULTS

### Data summary

Forty‐nine crocodiles were included in E1. Forty‐four crocodiles were alive at E2 (89.7%). Five crocodiles died between E1 and E2. The crocodile weights in E1 (excluding the crocodiles that died) ranged from 1.2 to 12.5 kg with a median of 4.5 kg (interquartile range: 3.1‒7.3 kg). The crocodile weights in E2 ranged from 1.1 to 16.8 kg with a median of 8 kg (interquartile range: 4.4‒12.5 kg). The change in weight of the crocodiles that survived ranged from ‒1.5 to +7.4 kg with a mean change in weight of +2.73 kg (SD 1.98). The crocodiles ranged from 83 to 167 cm in length during the entire study, with the largest growth in crocodile length of 40 cm between E1 and E2. Two crocodiles lost weight during the study; however, both of these crocodiles survived. The weights of the five deceased crocodiles ranged from 1.7 to 4.2 kg, with a median of 3.6 kg. Crocodiles that did not survive had a significantly lower initial weight compared to crocodiles that survived (*p* = 0.002).

The gastrointestinal tract was poorly defined in the radiographs acquired. The majority of gastroliths were positioned in the expected location of the stomach. However, occasional small gastroliths were visualised more caudally, likely within the small intestines or colon. Occasional accumulations of gas were identified in the stomach, colon and cloaca. There was no radiographic evidence of ingested metallic foreign material in the crocodiles in this study.

A total of 1971 gastroliths were recorded across the two examinations, with a mean of 21 gastroliths (SD 21) and a range of 0‒98 gastroliths. Excluding the deceased crocodiles, the total number of gastroliths recorded in E1 was 1198, with a minimum of 0, a maximum of 98 and a mean of 27.2 gastroliths (SD 27.2). Fewer gastroliths were recorded in E2 when compared with E1. The total number of gastroliths recorded in E2 was 750, with a minimum of 0, a maximum of 82 and a mean of 15.1 gastroliths (SD 15.1). Only two crocodiles (4.5%) in the study had no gastroliths in E1 and E2. Overall, 40.8% of the crocodiles had sand present in E1, which decreased to 8% in E2. The descriptive analysis of each relative gastrolith size, excluding the deceased crocodiles, is presented in Table 1. The five crocodiles that died between E1 and E2 had a total overall number of 31 gastroliths recorded in E1, with a minimum of one, a maximum of 10 and a median of seven gastroliths.

**TABLE 1:** Descriptive analysis results of the relative gastrolith sizes recorded (sand, small, medium and large) at examination 1 and examination 2.

Relative size of gastrolith	Descriptive analysis	Examination 1 (excluding deceased)	Examination 2	Examination 1 (deceased animals only)
Sand	Total	20	8	2
	Percentage	40.8%	18.6%	40%
Small gastroliths	Total overall	1191	674	28
	Minimum	0	0	1
	Maximum	98	80	10
	Mean	26.4	15.5	5.6
	Median	17	8	6
	Standard deviation	27.1	19.9	3.4
Medium gastroliths	Total overall	32	65	2
	Minimum	0	0	0
	Maximum	2	6	1
	Mean	0.5	1.5	0.4
	Median	0	1	0
	Standard deviation	0.76	1.8	0.6
Large gastroliths	Total overall	6	11	1
	Minimum	0	0	0
	Maximum	1	3	1
	Mean	0.1	0.3	0.2
	Median	0	0	0
	Standard deviation	0.3	0.6	0.5
All gastroliths	Total	1198	750	31

### Analysis models

**FIGURE 2:**
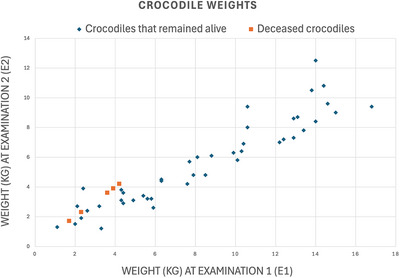
Scatter plot of the weights (kg) of the crocodiles that survived at examination 1 (E1) and examination 2 (E2). The crocodiles that were dead at E2 were plotted with the same weight at E1 and E2.

The smaller crocodiles failed to thrive between E1 and E2, with a reduced proportional bodyweight gain throughout the study, as demonstrated by a significant association between the E1 (start weight) and the change in weight (Figure 2) (*p* < 0.001). Larger crocodiles gained more weight in E2. A 1 kg increase in initial weight at E1 was associated with a 0.42 kg (95% confidence interval: [0.26–0.59]) increase in weight gain at E2. There was no significant association between the weight gain and the number of gastroliths at E1 (*p* = 0.506) or E2 (*p* = 0.2034).

**FIGURE 3:**
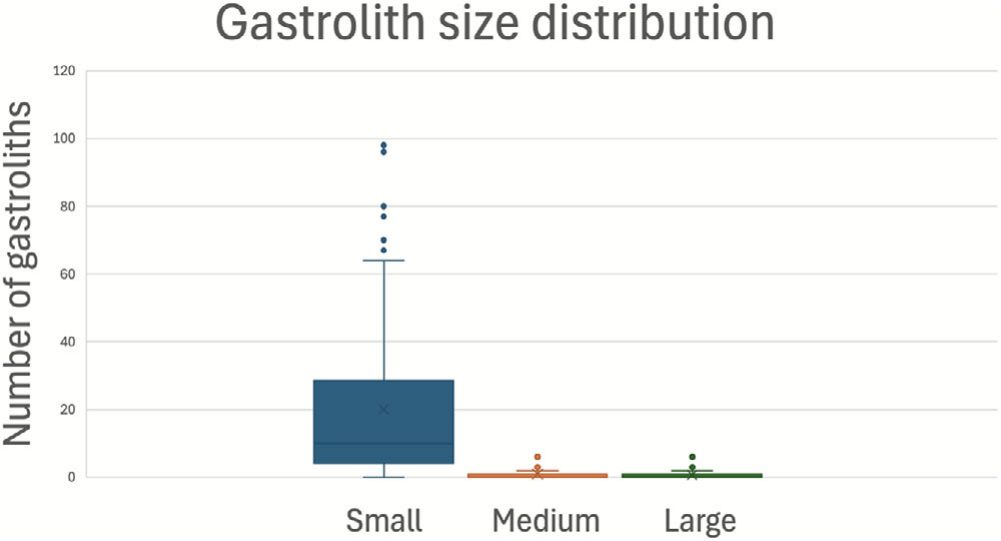
Histogram demonstrating the distribution of the relative size of gastroliths (small, medium or large) recorded during this study, combining the data from examination 1 and examination 2.

**FIGURE 4:**
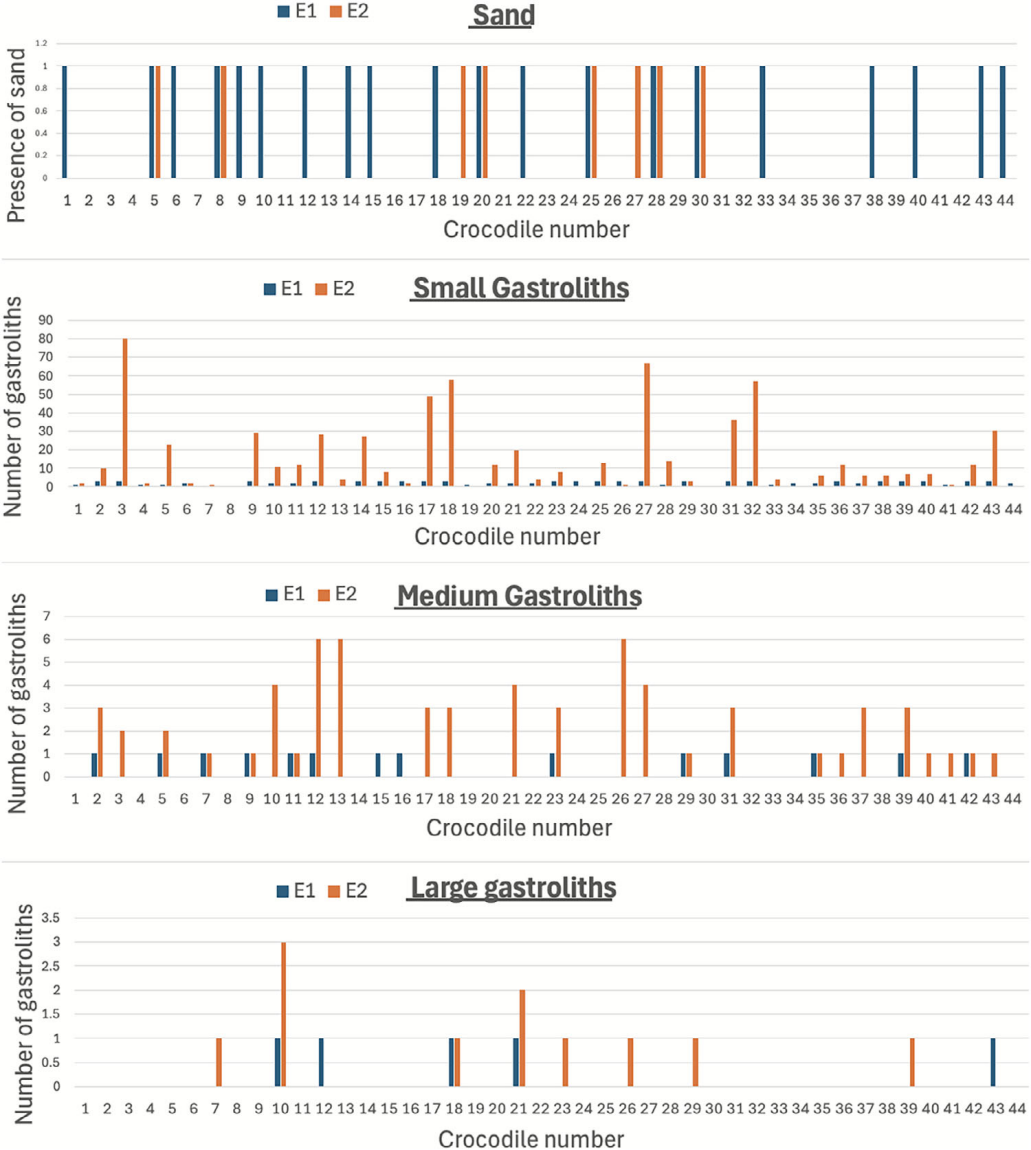
Histograms comparing the number of gastroliths of each relative size category (sand, small, medium and large) recorded for each crocodile in examination 1 (E1) and examination 2 (E2). Sand was recorded as either 1 (present) or 0 (absent).

**FIGURE 5:**
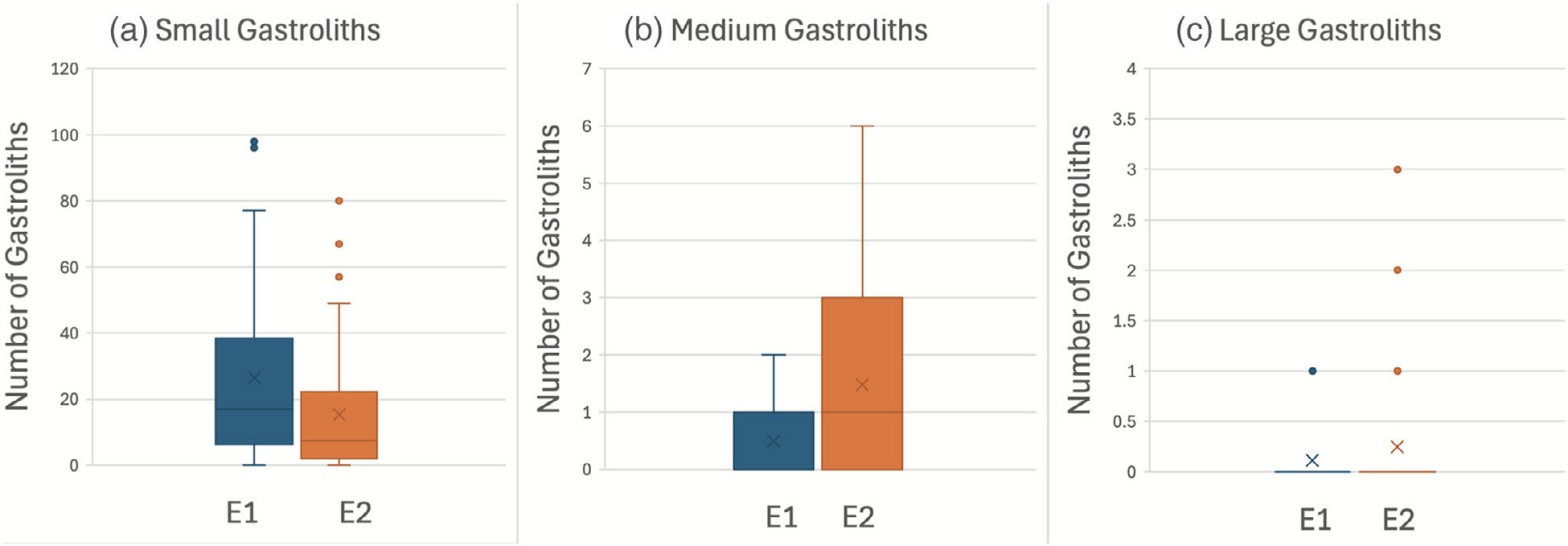
Histograms comparing the number of gastroliths recorded in each crocodile at examination 1 (E1) and examination 2 (E2), excluding deceased crocodiles. (a) Number of small gastroliths per crocodile; (b) number of medium gastroliths per crocodile; and (c) number of large gastroliths per crocodile.

Small gastroliths were the most common gastroliths in E1 and E2 (*p* < 0.001), with 94% of all gastroliths recorded being small (Table 2 and Figures 3 and 4). There was a significant decrease in the number of small gastroliths recorded per crocodile in E2 (mean 15.3) when compared with E1 (mean 26.4, *p* = 0.036) (Figure 5B). There was a significant increase in the number of medium gastroliths per crocodile in E2 (mean 1.5) when compared with E1 (mean 0.5, *p* = 0.002) (Figure 5C). The crocodiles that had medium/large stones in E1 were more likely to have medium/large stones in E2 (Table 1). There was a trend towards an increased number of large gastroliths recorded overall between E1 (*n* = 6) and E2 (*n* = 11); however, per crocodile, this was not statistically significant (*p* = 0.197), likely due to the small number of gastroliths in this category.

**TABLE 2:** Change in the largest gastrolith size between examination 1 (E1) and examination 2 (E2).

Largest gastrolith E1	Largest gastrolith E2	Number of crocodiles
Sand/none	Sand/none	3
Sand/none	Small	0
Sand/none	Medium/large	1
Small	Sand/none	3
Small	Small	11
Small	Medium/large	7
Medium/large	Sand/none	0
Medium/large	Small	2
Medium/large	Medium/large	17

*Note*: Crocodiles were categorised at E1 and E2 dependent on the largest gastrolith recorded on the radiograph. This table demonstrates how small, medium and large gastroliths are both ingested (shaded blue) and excreted (shaded grey). The crocodiles with medium/large stones in E1 were more likely to have medium/large stones in E2 (shaded pink).

When comparing the alive and the dead crocodiles at E1, the crocodiles that died had a significantly lower number of small gastroliths (*p* < 0.001) and total number of gastroliths (*p* < 0.001). There was no significant difference in the number of medium (*p* = 0.73) and large gastroliths (*p* = 0.69) between the dead and alive crocodiles. The crocodile with the largest gastrolith in this study died between E1 and E2.

### Pathology findings

Four crocodiles were found deceased in the enclosure during the 4‐month period between E1 and E2. One crocodile was humanely euthanased at E2 due to marked ocular lesions, wasted body condition and lethargy. The cohort had a survival rate of 90% over a 4‐month period. The weights of the deceased crocodiles ranged from 1.7 to 4.2 kg, with a mean of 3 kg. The crocodile that lost the most weight (1.5 kg) died after E2. One of the dead crocodiles was identified with the largest gastrolith relative to the size of the crocodile in the study (Figure 1D). However, postmortem examination data were not available for this crocodile. Postmortem examination of the remaining four crocodiles revealed no association between gastroliths and the cause of death. Gross postmortem findings included a combination of conjunctivitis and/or pharyngitis in three of the crocodiles. Multifocal 0.1‒0.3 cm white well‐demarcated lesions were found throughout the lung surface and parenchyma in the three crocodiles. The liver appeared enlarged and firm in two crocodiles. Multiple small non‐obstructive gastroliths were found in the two crocodiles. Histopathological evaluation confirmed the macroscopically appreciable granulomatous pneumonia in one crocodile. No fungal or acid‐fast bacterial organisms were identified in the tissue levels examined. *Chlamydia* and herpesvirus have subsequently been identified as likely causes. Histological evaluation of other organs was otherwise limited due to freezing artefact and postmortem autolysis.

The two examinations were timed to coincide with medical checks and movement of the crocodiles, in order to reduce handling time and stress. Blood samples (for haematology and biochemistry) were obtained by the zoo veterinarian from five crocodiles within this cohort at examination^2^ because of the slightly reduced appetite reported by the zoo keepers. There was no evidence of mineral deficiencies on these blood tests.

## DISCUSSION

Only two crocodiles (4.5%) in this study had no radiographic evidence of gastroliths in E1 and E2, demonstrating that lithophagy is an inherent behaviour in all crocodiles, consistent with previous literature. This is the first study to radiographically evaluate gastroliths in a large group of juvenile crocodiles. This study provides evidence that the presence of gastroliths in the coelomic cavity is a normal radiographic finding, when investigating crocodilian patients with suspected gastrointestinal disease.

The gastrointestinal tract was poorly defined in all radiographs acquired, a common finding in reptiles.^32^ This is likely due to the lack of internal fat between coelomic organs. No pneumocoelom was identified. Small amounts of gas were present in the expected location of the stomach, colon or cloaca (normal appearance). Orthogonal radiographs are recommended for the complete evaluation of the presence of pneumocoelom in reptiles presenting with gastrointestinal signs.^33^ Orthogonal radiographic projections enabling evaluation of the relative size of the gastroliths in the mediolateral orientation were not possible in this study. It was not considered appropriate for the handlers to manually restrain 49 crocodiles for the radiographic acquisition due to both ethical concerns of inducing unnecessary stress in the crocodiles by excessive handling and the concern of radiation exposure to the handlers. Orthogonal radiographic projections were not possible when the plexiglass box was used due to the integrity of the box, which contained plexiglass only on the top and bottom to ensure stability. The evaluation of only the dorsoventral radiographic projection may have resulted in some superimposition of gastroliths and miscounting of small gastroliths. The authors believe that these limitations were unlikely to significantly change the results or outcomes of this study but recommend that both projections are taken in clinical cases.

In this study, the number of gastroliths recorded per crocodile varied considerably with a range of 0‒98 gastroliths per crocodile and a mean of 21 gastroliths (SD 21). The crocodiles with large numbers of small gastroliths had no clinical signs of gastrointestinal disease; therefore, this study provides evidence that large numbers of small gastroliths, relative to the crocodile's size, are considered a normal radiographic finding. Small gastroliths, defined as less than 0.5 × length of the D11 vertebrae, were by far the most common gastroliths recorded in E1 and E2 (Table 2), representing 94% of all gastroliths.

The gastroliths in this study were mostly found in the region of the stomach, with very few congregations found caudally to the stomach, likely retained by the action of a well‐developed pyloric sphincter. Previous studies have demonstrated the dynamic ingestion, gastric dispersion and excretion of boluses of different food substrates in juvenile crocodiles.^15^, ^17^, ^19^ There was a significant decrease in the total number of gastroliths recorded per crocodile between E1 and E2 (*p* < 0.001), predominantly due to a decrease in the amount of small gastroliths recorded per crocodile (*p* = 0.034), which were likely excreted (Figure 5B). It is also considered possible that the gastroliths may have increased in size by accumulating more mineral material. The crocodiles that had medium/large stones in E1 were more likely to have medium/large stones in E2 (Table 1). These findings suggest that although crocodiles can excrete small, medium and large gastroliths (Table 1), they are more likely to retain medium/large gastroliths.

Gastroliths are found within a variety of fossil and living animals, such as seals, whales and frog tadpoles.^34^, ^35^, ^36^ The reason for intentional lithophagy in crocodilian species has been widely discussed, with mixing of foodstuff, mineral supply, stomach cleaning, alleviation of hunger, ballast (stability in the water) and accidental ingestion all proposed as possible hypotheses for cause of lithophagy.^34^, ^35^ More recent evidence suggests that lithophagy may have an important role in moderating the crocodile's buoyancy to allow increasing lung volumes and oxygen storage to extend diving times for hunting.^37^, ^38^ This study found increasing numbers of medium and large gastroliths in E2 (Figure 5C). This finding is consistent with a previous study that performed gastric lavage in wild crocodiles and found an increase in total gastrolith mass with increasing bodyweight and age.^15^ As the juvenile crocodile's diet changes from insects to invertebrates and larger prey, they may ingest larger gastroliths to reduce buoyancy and increase diving time.^37^

The five crocodiles that died between E1 and E2 demonstrated significantly decreased bodyweight (range: 1.7‒4.2 kg, median: 3.6 kg) when compared with the crocodiles that survived (*p* = 0.002). Two other crocodiles in this study lost weight between examinations, which might represent limitations of the weighing equipment to detect small changes in weight or represent wasting associated with infectious disease or ‘runting’, a known problem in farmed saltwater crocodiles.^11^ The five crocodiles that died between E1 and E2 had decreased total number of gastroliths (*p* < 0.001) and small gastroliths recorded (*p* < 0.001) in the radiographs acquired at E1, when compared to the crocodiles that remained alive. There was no significant difference in the number of medium (*p* = 0.72) and large gastroliths (*p* = 0.69) between the alive and deceased crocodiles, which may be due to the small number of medium/large gastroliths and the small number of deceased crocodiles. The presence of relatively small gastroliths within the gastrointestinal tract may be seen as evidence of a good appetite; therefore, the lack of them may be considered a poor prognostic indicator in unwell crocodilian patients.

The crocodile with the largest gastrolith in this study (Figure 1D) died between E1 and E2. Unfortunately, no postmortem examination was available for this crocodile; therefore, the cause of death remains unknown. Previous necropsies of crocodiles from other cohorts in the same unit have found coelomitis secondary to large or sharp objects. Although it is considered possible that the size of the gastrolith in this crocodile, measuring three times the length of the D11 vertebral body, may have been implicated in the cause of death, this is entirely speculative. At the time of the study, it was not known if this was a problem, as the crocodile had no clinical signs of being unwell; however, in the future, more careful monitoring of patients with gastroliths of this relative size is advised. Further research regarding maximal gastrolith size relative to the size of the crocodile and evidence of gastrointestinal obstruction may be considered for future studies to guide veterinarians in this field.

A limitation of this study is that all radiographs were performed on a cohort of juvenile crocodiles at the same location from a single age group due to safety concerns related to catching and handling a group of large conscious adult crocodiles. The specific findings of this study may not be entirely representative of adult crocodilians, wild crocodilians or crocodilians in other farm establishments. However, the authors feel that the general trends may be safely extrapolated due to previously published research including these age categories.^15^

The crocodiles grew in length during the study between E1 and E2, with the largest total increase in length (from nose to tail) of 40 cm in one crocodile. In this crocodile, the length of the 11th dorsal vertebral body radiographically increased from 1.8 to 2.1 cm, with a difference of 3 mm. This may have resulted in re‐classification of larger gastroliths as medium or small gastroliths in E2. Although this is possible, the results demonstrate the opposite trend, with increasing numbers of medium/large gastroliths. Therefore, although this is accepted as a limitation, it is unlikely that this significantly impacted the results overall.

Only one radiologist resident evaluated all of the radiographs in this study, supervised by a boarded radiologist who checked a random selection of radiographs and agreed with the resident's evaluation. Inter‐observer and intra‐observer statistical evaluations were beyond the scope of this study. Multiple observers may have led to slightly different results when counting large numbers of small gastroliths, which is acknowledged as a limitation of this study.

## CONCLUSION

The concept of gastroliths in crocodiles is not novel in reptile medicine, however, this study provides valuable guidance to veterinarians when interpreting radiographs of individual patients in captivity presenting with gastrointestinal signs. Large amounts of relatively small gastroliths (<0.5 × the length of the D11 vertebral body) are recognised as a normal radiographic finding in juvenile crocodiles, providing evidence of a good appetite. As crocodiles increase in age and size, it is considered likely they retain and ingest more medium/large gastroliths.^32^ Patients with relatively large gastroliths measuring over three times a vertebral body in length should be carefully monitored for signs of being unwell or gastrointestinal obstruction. This study demonstrates the inherent lithophagy compulsion of crocodilians in captivity and highlights the importance of careful management of the environmental to prevent ingestion of sharp, metallic or large ingestible mineral material.

## AUTHOR CONTRIBUTIONS

*Concept and design*: Charlotte R. Nix and Marie‐Aude Genain. *Acquisition of data*: Charlotte R. Nix, Iain Cope and Marie‐Aude Genain. *Analysis and interpretation of data*: Charlotte R. Nix, Ellie R. John, Katherine Hughes and Marie‐Aude Genain. *Drafting the article*: Charlotte R. Nix and Katherine Hughes. *Reviewing the article for intellectual content*: Charlotte R. Nix, Ellie R. John, Katherine Hughes, Iain Cope and Marie‐Aude Genain. All the authors approved the final article and agree to be accountable for all aspects of the work.

## CONFLICT OF INTEREST STATEMENT

The authors declare they have no conflicts of interest.

## FUNDING INFORMATION

The authors received no specific funding for this study.

## ETHICS STATEMENT

The authors confirm that the ethical policies of the journal, as noted on the journal's author guidelines page, have been adhered to. The research was approved by the Departmental Ethics and Welfare Committee at the Department of Veterinary Medicine, University of Cambridge (CR672).

## Data Availability

The data that support the findings of this study are available from the corresponding author upon reasonable request.
